# Association between epithelial‐mesenchymal transition and cancer stemness and their effect on the prognosis of lung adenocarcinoma

**DOI:** 10.1002/cam4.556

**Published:** 2015-10-16

**Authors:** Terumasa Sowa, Toshi Menju, Makoto Sonobe, Takao Nakanishi, Kei Shikuma, Naoto Imamura, Hideki Motoyama, Kyoko Hijiya, Akihiro Aoyama, Fengshi Chen, Toshihiko Sato, Masashi Kobayashi, Akihiko Yoshizawa, Hironori Haga, Takashi Sozu, Hiroshi Date

**Affiliations:** ^1^ Department of Thoracic SurgeryGraduate School of MedicineKyoto UniversityKyotoJapan; ^2^ Department of Thoracic SurgeryTokyo Medical and Dental UniversityTokyoJapan; ^3^ Department of Diagnostic PathologyKyoto University HospitalKyotoJapan; ^4^ Department of Management ScienceFaculty of EngineeringTokyo University of ScienceTokyoJapan

**Keywords:** Cancer stemness, CD133, epithelial‐mesenchymal transition, lung adenocarcinoma, vimentin

## Abstract

The epithelial‐mesenchymal transition (EMT) and cancer stemness (CS) are reported to be pivotal phenomena involved in metastasis, recurrence, and drug‐resistance in lung cancer; however, their effects on tumor malignancy in clinical settings are not completely understood. The mutual association between these factors also remains elusive and are worthy of investigation. The purpose of this study was to elucidate the association between EMT and CS, and their effect on the prognosis of patients with lung adenocarcinoma. A total of 239 lung adenocarcinoma specimens were collected from patients who had undergone surgery at Kyoto University Hospital from January 2001 to December 2007. Both EMT (E‐cadherin,vimentin) and CS (CD133, CD44, aldehyde dehydrogenase) markers were analyzed through immunostaining of tumor specimens. The association between EMT and CS as well as the patients' clinical information was integrated and statistically analyzed. The molecular expression of E‐cadherin, vimentin, and CD133 were significantly correlated with prognosis (*P* = 0.003, *P* = 0.005, and *P* < 0.001). A negative correlation was found between E‐cadherin and vimentin expression (*P* < 0.001), whereas, a positive correlation was found between vimentin and CD133 expression (*P* = 0.020). CD133 was a stronger prognostic factor than an EMT marker. Elevated CD133 expression is the signature marker of EMT and CS association in lung adenocarcinoma. EMT and CS are associated in lung adenocarcinoma. Importantly, CD133 is suggested to be the key factor that links EMT and CS, thereby exacerbating tumor progression.

## Introduction

Lung cancer has been the leading cause of cancer death worldwide 1, and adenocarcinoma is the most common histologic subtype of primary lung cancer 2. Recently, the therapeutic strategies for lung cancer have been developing and shifting from cytotoxic reagents to molecular‐targeted reagents. However, the evolving mechanisms through which cancer cells are resistant to these drugs have emerged as some of the most challenging properties of cancer to overcome. Epithelial‐mesenchymal transition (EMT) and cancer stemness (CS) are known to be pivotal for driving metastasis, recurrence, and resistance for treatment in lung cancer, but the nature of these factors is not completely understood.

EMT is a critical event not only in embryonic development and in cell migration during wound healing or tissue development 3, 4, but also in the migration of malignant cells undergoing invasion and metastasis 4, 5, 6. EMT is composed of two processes: the loss of epithelial characters and the gain of mesenchymal characters 4, 7. E‐cadherin is the membrane protein working as an adhesion molecule, and it is the most common epithelial factor. The loss of E‐cadherin is one of the major features of EMT 4. In contrast, N‐Cadherin 8, SNAIL 4, 9, TWIST 10, and vimentin 4, 5, 11, 12 are mesenchymal factors that work during various phases of EMT. Vimentin, a member of the intermediate filament family, is the protein responsible for maintaining cellular integrity and reducing damage caused by stress. Vimentin is expressed in normal mesenchymal cells, and recent studies have shown a correlation between increased levels of vimentin expression and malignant progression in various types of cancers, such as prostate, gastrointestinal, central nervous system, breast, malignant melanoma, and lung cancers 12.

On the other hand, the cancer stem cell was first reported in 1997 by Bonnet, in human acute myeloid leukemia 13. The presence of cancer stem cells in solid tumors, such as breast cancer 14, colon cancer 15, pancreatic cancer 16, and brain tumors 17, has been proposed recently. Cancer stem cells are also called cancer‐initiating cells because of their capacities for self‐renewal, multi‐lineage differentiation, and higher levels of malignancy 18, 19. Some studies have shown that cancer stem cells are resistant to therapy and are responsible for tumor recurrence and metastasis 20. There are many reports in which CS markers correlated with patients' prognoses. Previous reports demonstrated that CD133 expression is correlated with brain 21 and colon cancer 22, CD44 is correlated with pancreatic cancer 23, and aldehyde dehydrogenase (ALDH) is correlated with ovarian cancers 24. Some reports mentioned the presence of CS markers in lung cancer 25, 26, 27, but they are not definitive yet.

Previously, EMT and CS have been separately reported in terms of their unfavorable effects on prognosis. In in vitro experiments, the associations among each property have been reported 28, whereas their mutual associations in a clinical setting remain elusive.

The purposes of our present study are to elucidate the association between the EMT and CS and to determine their effect on the prognosis in lung adenocarcinoma. We also present the key factors that link EMT and CS and discuss how these factors affect tumor malignancy in this study.

## Methods

### Patient selection

A total of 239 specimens of lung adenocarcinomas were collected from patients who had undergone surgery at the Kyoto University Hospital from January 2001 to December 2007. Patients who received neoadjuvant therapy, underwent incurable surgery, or had multiple cancers, were excluded. All specimens were subjected to tissue microarray (TMA) analysis, as described below. The tumors were staged according to the 7th edition of the TNM classification of the International Union Against Cancer 29. Histological classification was according to the 2004 WHO classification 30 and the IASLC/ATS/ERS classification of lung adenocarcinoma 31. According to the IASLC/ATS/ERS criteria, each tumor was reviewed using comprehensive histologic subtyping and the percentage of each histologic component in 5% increments was recorded. The survival time and outcome data were available for all 239 patients, with a median follow‐up time of 63.0 months (range: 1–129 months). Approval for the use of the tissues in this research was obtained from the Institutional Review Board of Kyoto University.

### Tissue microarray

TMAs were made by the pathologists in the department of Diagnostic Pathology in Kyoto University Hospital 32 using a similar approach to that described previously by Kononen et al. 33. Briefly, after the case selection described above, paraffin‐embedded tumor blocks with sufficient tissue were selected for TMA. The most representative regions of the tumors were selected based on the morphology of the H&E‐stained slide. Tissue cores measuring 2 mm in diameter were punched out from each donor tumor block, using thin‐walled stainless steel needles (Azumaya Medical Instruments Inc., Tokyo, Japan), and were arrayed in a recipient paraffin block. Non‐neoplastic lung tissue cores from selected patients were also arrayed in the same block to serve as negative controls.

### Immunohistochemical analysis

**Figure 1:**
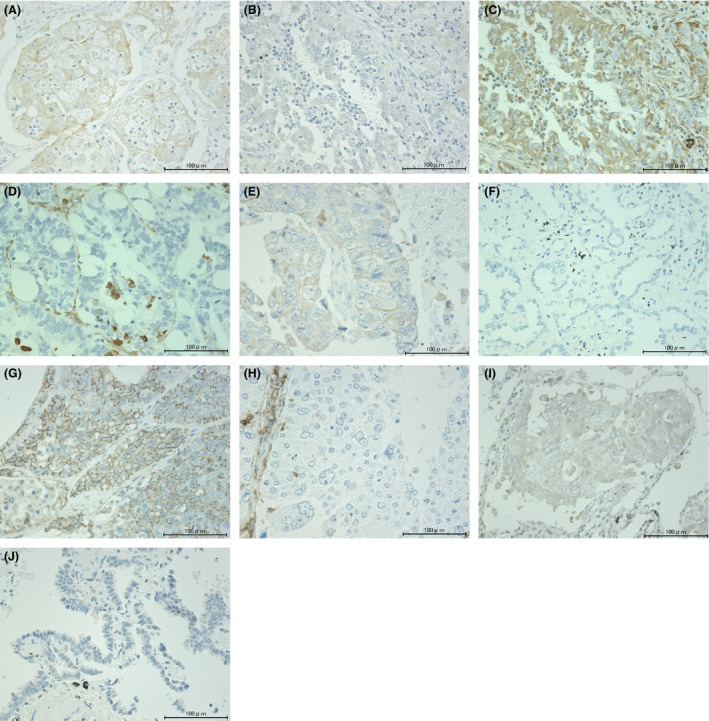
Representative images of immunohistochemical staining of lung adenocarcinoma sections with anti‐E‐cadherin (A and B), vimentin (C and D), CD133 (E and F), CD44 (G and H), and ALDH (I and J) antibodies. (A, C, E, G, and I) show positive expression, while B, D, F, H, and J show negative expression. (Original magnification, ×400). ALDH, aldehyde dehydrogenase.

A standard immunostaining technique, which was previously published 34, was implemented in this study. Immunostaining against E‐cadherin, vimentin, CD133, CD44, and ALDH was performed with mouse anti‐human E‐cadherin monoclonal antibody (36B5, dilution 1:300, Leica Biosystems, Newcastle, UK), mouse anti‐human vimentin monoclonal antibody (SRL33, dilution 1:300, Leica Biosystems), mouse anti‐human CD133 monoclonal antibody (W6B3C1, dilution 1:10, Miltenyi Biotec, Auburn, CA), mouse anti‐human CD44 monoclonal antibody (156‐3C11, dilution 1:50; Cell Signaling Technology, Danvers, MA), and rabbit anti‐human ALDH1A1 polyclonal antibody (dilution at 1:3000, Abcam, Cambridge, UK). After deparaffinization and rehydration, the slides were heated in a microwave for 20 min in 10 *μ*mol/L citrate buffer solution at pH 6.0 (E‐cadherin, vimentin, CD133, CD44), or heated by autoclaving for 5 min (ALDH). After quenching the endogenous peroxidase activity with 0.3% H_2_O_2_ in absolute methanol for 30 min, the sections were treated with 1% horse (E‐cadherin, vimentin, CD133, CD44) or goat (ALDH) normal serum albumin. The sections were incubated overnight with the primary antibodies. Slides were then incubated for 1 h with each equivalent biotinylated secondary antibody. The sections were incubated with the avidin–peroxidase complex for 1 h and visualized with 3,3′‐diaminobenzidine tetrahydrochloride (Dojindo laboratories, Kumamto, Japan). Lastly, the sections were lightly counterstained with Mayer's hematoxylin. The immunostained sections were examined by two authors (T. S. and T. M.) without knowledge of the patient characteristics. Cases with discrepancies were jointly reevaluated until a consensus was reached. After immunostaining, the expressions of these proteins were examined in four distinct fields with a minimum of 500 cells. The proportion of positive cells was measured and classified as 0 (no staining), +1 (weak), +2 (moderate), and +3 (strong), and each specimen was categorized as negative (0, +1) or positive (+2, +3). Figure 1 shows representative images of immunohistochemical staining by anti E‐cadherin (A, B), vimentin (C, D), CD133 (E, F), CD44 (G, H), and ALDH (I, J) antibodies.

### Statistical analysis

Age and average smoking index were summarized using the mean ± SD, whereas the categorical variables were summarized using counts and percentages. The OS (overall survival) was calculated from the date of surgery. Time‐to‐event curves for OS were estimated using the Kaplan–Meier method, and differences in time‐to‐event curves were evaluated with the log‐rank test. The correlations among the five markers were analyzed using odds ratio and chi‐square tests. The analysis of the hazard ratio was performed using the Cox proportional hazards regression analysis. All P‐values were two‐sided and *P*‐values <0.05 were considered statistically significant. All statistical analyses were performed using SAS version 9.3 (SAS Institute Inc., Cary, NC).

## Results

### Clinicopathological characteristics

The clinicopathological characteristics of the patients and tumors evaluated in our study are summarized in Table 1. There were 123 (51.5%) men, with a mean age of 67.0 ± 9.6 years (range, 23–86 years). One hundred and nine patients (45.6%) had never smoked, and 130 patients (54.3%) were former or current smokers, with an average smoking index of 26.5 ± 36.4 pack‐years. The numbers of patients at each pathological stage were as follows: IA, 119 (49.8%); IB, 70 (29.3%); IIA, 22 (9.2%); IIB, 4 (1.7%); IIIA, 23 (9.6%) patients; and IIIB, 1 (0.4%) (Table 1). There were 189 (79.1%) pathological stage I cases in this study, which is higher than that generally reported. The frequency of EMT or CS marker expressions is shown in Table 2. The analysis of TMA specimens for EMT and CS markers indicated that of the 239 cases, 120 (50.2%) specimens were positive for E‐cadherin, 50 (20.9%) for vimentin, 26 (10.9%) for CD133, 48 (20.1%) for CD44, and 89 (37.2%) for ALDH, respectively.

**Table 1:** Characteristics of the patients included in this study

Variables	*N* = 239
Sex, *n* (%)	
Male	123 (51.5)
Female	116 (48.5)
Age (years), 67.0 ± 9.6 years old	
Smoking status	
Never	109 (45.6%)
Former/current	130 (54.3%)
Average smoking index	26.5 ± 36.4 pack‐years
p‐Stage (%)	
IA	119 (49.8)
IB	70 (29.3)
IIA	22 (9.2)
IIB	4 (1.7)
IIIA	23 (9.6)
IIIB	1 (0.4)
Tumor grade (%)	
Well differentiated	47 (19.7)
Moderately differentiated	97 (40.6)
Poorly differentiated	95 (39.8)
Lymphatic invasion (%)	
Absent	193 (80.8)
Present	46 (19.3)
Vascular invasion (%)	
Absent	184 (77.0)
Present	55 (23.0)
Pleural invasion (%)	
pl 0	188 (78.7)
pl 1‐3	51 (21.3)
IASLC/ATS/ERS classification of lung adenocarcinoma (%)	
Adenocarcinoma in situ	9 (3.8)
Minimally invasive adenocarcinoma	13 (5.4)
Lepidic predominant	18 (7.5)
Acinar predominant	31 (13.0)
Papillary predominant	111 (46.4)
Micropapillary predominant	8 (3.4)
Solid predominant	41 (17.2)
Invasive mucinous adenocarcinoma	7 (2.9)
Others	1 (0.4)

**Table 2:** Expressions of EMT and CS markers in the specimens

	E‐cadherin	Vimentin	CD133	CD44	ALDH
Positive	120 (50.2%)	50 (20.9%)	26 (10.9%)	48 (20.1%)	89 (37.2%)
Negative	119 (49.8%)	189 (79.1%)	213 (89.1%)	191 (79.9%)	149 (62.3%)

One specimen could not be evaluated for its ALDH expression, because the specimen was insufficient in the tissue microarray slide. EMT, epithelial‐mesenchymal transition; CS, cancer stemness; ALDH, aldehyde dehydrogenase.

### Relationship between EMT markers and patient prognosis

**Figure 2:**
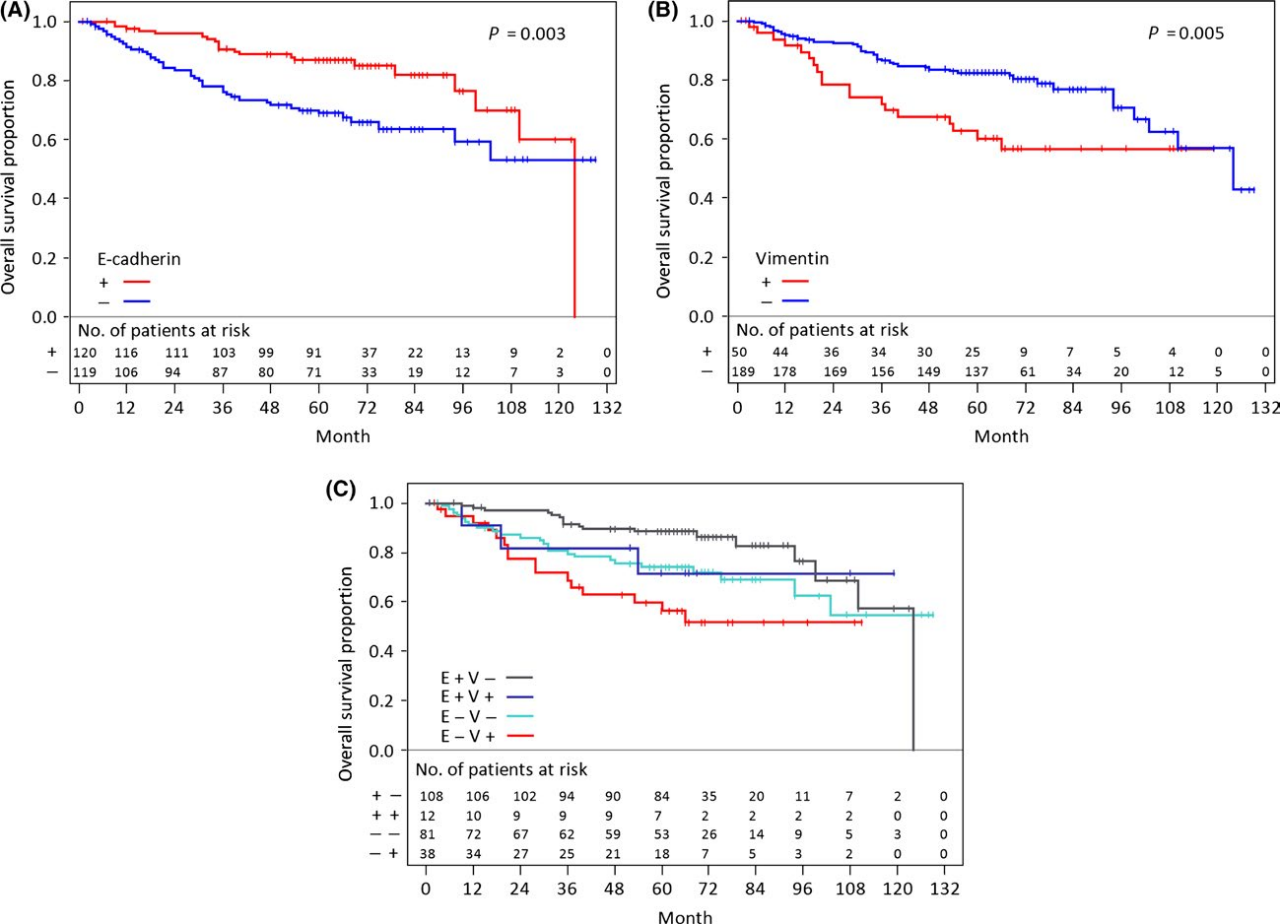
Kaplan–Meier curves for overall survival and log‐rank *P* values according to EMT markers. (A) E‐cadherin, (B) vimentin, and (C) combination of EMT markers. (E+) E‐cadherin positive, (E−) E‐cadherin negative, (V+) vimentin positive, and (V−) vimentin negative, EMT, epithelial‐mesenchymal transition.

The prognoses of patients depending on EMT markers are shown in Figures 2A and B. The negative E‐cadherin group and the positive vimentin group had significantly poorer prognoses (Fig. 2A and B, *P* *=* 0.003 and *P = *0.005*,* respectively). The prognoses of patients depending on the combination of EMT markers are shown in Figure 2C. These data suggest that the null EMT conversion group (positive E‐cadherin and negative vimentin) had the best prognosis, and that patients with EMT progression indicated a worse prognosis.

### Relationship between CS markers and patient prognosis

**Figure 3:**
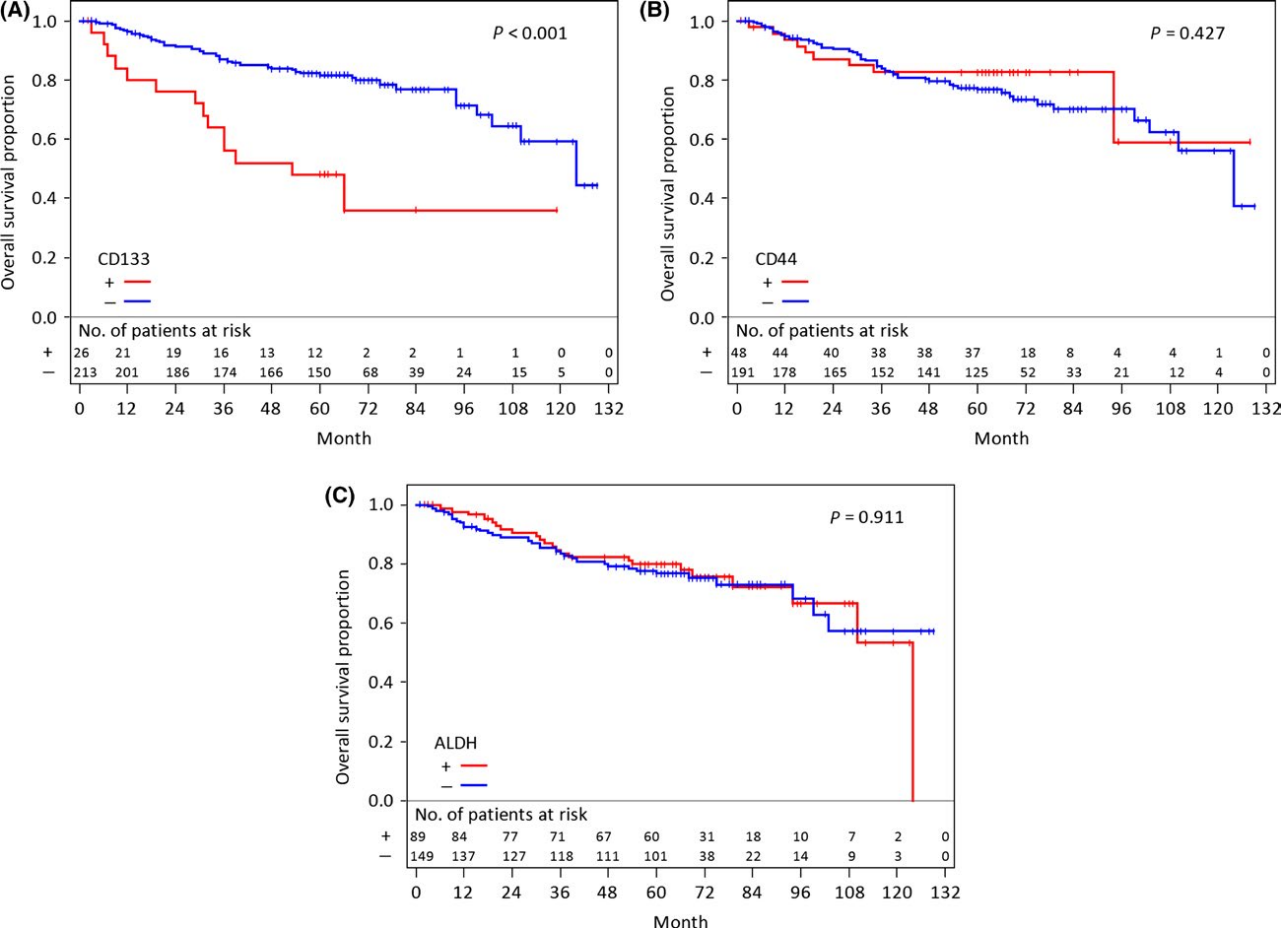
Kaplan–Meier curves for overall survival and log‐rank *P* values according to CS markers. (A) CD133, (B) CD44, and (C) ALDH. ALDH, aldehyde dehydrogenase; CS, cancer stemness.

The prognoses affected by CS markers are shown in Figure 3A, B, and C. The expression of CD133 had a significantly unfavorable effect on prognosis (Fig. 3A, *P *<* *0.001). However, the expression of CD44 and ALDH did not significantly correlate with the prognosis of the patients (Fig. 3B and C, P = 0.427 *and P *=* *0.911, respectively). In our study, CD133 is the only prognostic factor of CS among these markers.

### Association among EMT and CS markers in lung adenocarcinoma

**Figure 4:**
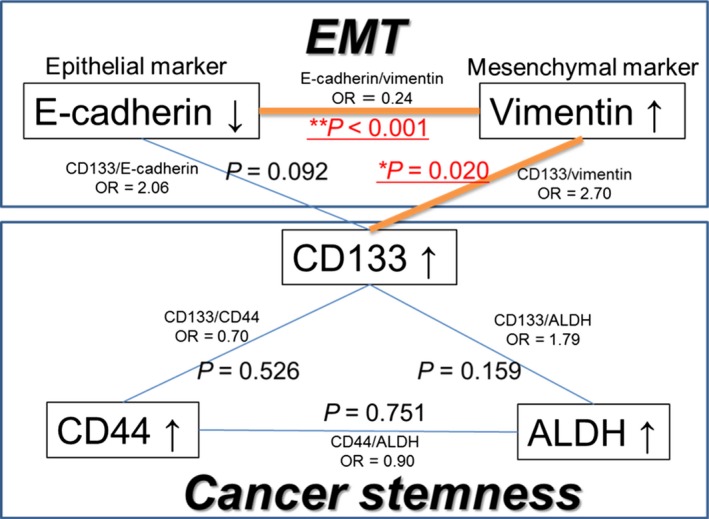
Association among EMT and CS markers. EMT, epithelial‐mesenchymal transition; CS, cancer stemness; ALDH, aldehyde dehydrogenase; OR, odds ratio.

The association between EMT and CS markers is shown in Figure 4. A negative correlation was found between E‐cadherin and vimentin expression (*P *<* *0.001), whereas, a positive correlation was found between vimentin and CD133 expression (*P *=* *0.020). Only CD133 was significantly correlated with EMT markers rather than the other CS markers.

The expressions of the EMT marker vimentin and the CS marker CD133 showed significant correlation, suggesting that these factors are the key factors linking EMT and CS.

### Effects of EMT and CS markers on patient prognosis of lung adenocarcinoma in multivariate analysis

We analyzed the effects of the expressions of CD133 and EMT markers on OS. Because CD133 was the strongest prognostic marker, as shown in Figure 3A, we divided the patients into positive or negative CD133 groups. In the positive CD133 group, there were no clear differences in the Kaplan–Meier curves for OS among the subgroups with EMT progression. Since only 26 patients were included in this group, we combined them into one group for an additional analysis (Table 3, CD133+). Regarding the negative CD133 group, we classified this group into three groups, based on the expression of E‐cadherin and vimentin, as follows: full EMT conversion group, E‐cadherin‐negative and vimentin‐positive; partial EMT conversion group, both E‐cadherin‐ and vimentin‐negative or positive; and null EMT conversion group, E‐cadherin‐positive and vimentin‐negative.

**Table 3:** The Cox proportional hazards regression analysis for EMT and CS markers (adjustment factors: p‐stage, sex, age)

Parameter	Hazard ratio	95% CI	*P*‐value
CD133^+^	3.56	[1.62–7.81]	0.002
CD133− & Full EMT	1.90	[0.83–4.32]	0.127
CD133− & Partial EMT	2.07	[1.04–4.24]	0.038
CD133− & Null EMT (Reference)	1		
p‐stage [II, III/I]	6.38	[3.61–11.4]	<0.001
Sex [male/female]	1.58	[0.92–2.80]	0.099
Age	1.03	[1.00–1.06]	0.073

The hazard ratios of groups stratified by CD133 expression and EMT progression are shown in Table 3. Since only 26 patients were included in the CD133 positive group, we combined them into one group for this analysis (CD133+). These hazard ratios are standardized with that of the CD133− and null EMT conversion group. CI, confidence interval; EMT, epithelial‐mesenchymal transition; CS, cancer stemness.

**Figure 5:**
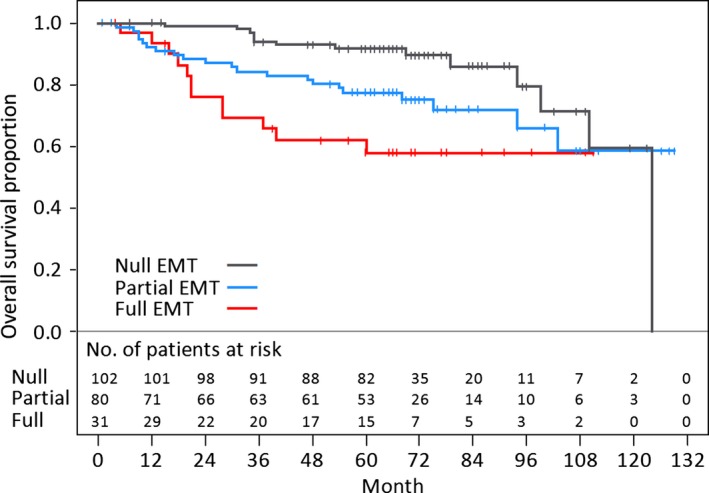
Kaplan–Meier curves for overall survival of the groups of CD133‐negative patients divided by the progression of EMT. Three groups of CD133‐negative patients are shown based on the expression of E‐cadherin and vimentin as follows; full EMT conversion group, E‐cadherin‐negative and vimentin‐positive; partial EMT conversion group, both E‐cadherin‐ and vimentin‐negative or positive; null EMT conversion group, E‐cadherin‐positive and vimentin‐negative. EMT, epithelial‐mesenchymal transition.

In Figure 5, there were clear differences in the Kaplan–Meier curves for OS among the negative CD133 groups divided by the progression of EMT. The group indicating negative CD133 and null EMT conversion showed the best prognosis.

Table 3 shows the results of a Cox regression analysis adjusted for covariates (p‐stage, sex, and age). As compared with the negative CD133 and null EMT conversion group, the hazard ratios (*P*‐values) of the positive CD133 group, negative CD133 and full EMT conversion group, and negative CD133 and partial EMT conversion group were 3.56 (*P *=* *0.002), 1.90 (*P *=* *0.127), and 2.07 (*P *=* *0.038), respectively. The positive CD133 group showed the worst prognosis (*P *=* *0.002).

## Discussion

Importantly, our research shows the effects of EMT and CS on patients' prognoses and a clear association between EMT and CS in clinical specimens. The EMT marker vimentin and CS marker CD133 will be focused on as the key factors linking EMT with CS.

EMT and CS have been reported to affect migration, invasion, metastasis, and therapeutic resistance 4, 6, 20. Surprisingly, there are few reports referring to the association of EMT and CS. In an in vitro study, Mani et al. reported that EMT‐induced normal epithelial mammary cells obtained the characteristics of CS including CD133 expression 28. Pirozzi et al. also reported that the EMT‐induced cancer cell line A549 acquired CS 35. In clinical reports, there have been many articles about each marker, but the associations between EMT and CS have not been reported previously. Our study revealed that vimentin and CD133 expression are correlated, and that the CS marker CD133 is associated with a worse prognosis than EMT conversion.

However, it remains unclear how vimentin and CD133 affected the prognoses in our report. CD133 is a five‐transmembrane cell surface glycoprotein family, and its gene, *PROM1* is specifically located on chromosome 4p15, a region that contains genes related to mature organ homoeostasis, tumorigenesis, and cancer progression 36. Previous studies have determined that positive CD133 cancer cells possess CS 36, but its precise function remains unclear. Tirino et al. investigated the role of CD133 by analyzing the differences between positive and negative CD133 subpopulations in the lung cancer cell line A549 37. The positive CD133 subpopulation expressed vimentin more strongly and had more potential for invasion, migration, and distant metastasis than the negative CD133 one. These data are compatible with our results; that is, there is correlation between the expression of CD133 and vimentin, and the group with positive CD133 expression had a worse prognosis. Although the major roles of CD133 remain unidentified, we have shown that CD133 has an important role in tumor progression in lung adenocarcinoma.

EMT progression was not an independent prognostic marker in our multivariate analysis (Table 3). EMT progression (full/partial/null) was significantly correlated with pathological stage (stage I/II, III) in our study (data not shown). This fact might have some effects on this result. Conversely, it is suggested that EMT correlates with T or N factors, which is very interesting.

Cancer, including lung adenocarcinoma, has been difficult to treat. The characteristics of the EMT and CS have been widely investigated, but EMT and CS have not been examined enough as therapeutic targets. Since the expressions of vimentin and CD133 are correlated, targeting CS via EMT may be possible. For example, silibinin was reported to inhibit tumor progression via vimentin and MMP‐2 suppression 38, 39, and salinomycin was reported to lead to the regression of cancer via the suppression of the EMT and CS marker, CD133 40. Our study supports these previous reports and shows the possibility of their application for clinical therapy.

It is necessary to understand the common background mechanisms underlying EMT and CS. The reports that transforming growth factor beta‐induced EMT and CS in an in vitro study 28, 35 suggest the probability that the tumor microenvironment including cancer–associated fibroblasts influence the EMT and CS. In addition, hypoxia is also reported to induce EMT and CS via the upregulation of hypoxia‐inducible factor 1*α* expression 41, 42. These mechanisms may underlie the results of the present study. Interventions targeting these factors will be necessary for the innovative therapy of lung cancer. Moreover, the associations of EMT or CS with pathological characteristics or genetic alternations are not definitive in lung cancer 43, 44. The analyses of these factors are now underway in our group, and they will also be useful to unveil the nature of EMT and CS.

There are some limitations to our study. First, the proportion of stage I cases in our study is 79.1%, and it is larger than what has generally been reported 45. This is because patients receiving neoadjuvant therapy, undergoing incurable surgery, and those with multiple cancers were excluded to ensure accurate evaluation of the association between these biomarkers and prognoses. It is possible that this fact had some effect on our results. Second, the expression of CD44 was detected using a mouse anti‐human CD44 monoclonal antibody (pan‐CD44), and it was not prognostic in our study. Regarding CD44, both pan‐CD44 and its variant CD44v6 were reported to be prognostic 26. It is not yet definitive which splice variant is more prognostic, and further investigation will be needed.

In conclusion, we showed that the progression of EMT and the expression of the CS marker CD133 had significantly unfavorable effects on the prognoses of lung adenocarcinoma patients. The EMT and CS were significantly correlated through vimentin and CD133. The CS marker CD133 was a stronger prognostic factor than the EMT markers. Further research into these factors is expected to elucidate how cancers acquire metastatic potency and resistance against treatments associated with the EMT and CS.

## Conflict of Interest

None declared.
